# Mechanical Circulatory Support in Congenital Heart Disease

**DOI:** 10.3390/children12030306

**Published:** 2025-02-28

**Authors:** Áine Lynch, Aamir Jeewa

**Affiliations:** 1 Division of Pediatric Cardiology, Columbia University Medical Center, New York Presbyterian Morgan Stanley Children’s Hospital, New York, NY 10032, USA; 2 Division of Cardiology, Department of Pediatrics, The Hospital for Sick Children, Toronto, ON M5G 1X8, Canada; aamir.jeewa@sickkids.ca

**Keywords:** mechanical circulatory support, ventricular assist device, pediatric heart failure, congenital heart disease

## Abstract

As early survival outcomes have improved, heart failure in children with congenital heart disease (CHD) has become a growing problem. Primary care providers and pediatricians are thus encountering increasing numbers of children with or at-risk for heart failure. Despite medication, many of these children progress to end-stage heart failure and require heart transplant for long-term survival. Mechanical circulatory support (MCS) is increasingly utilized to support this cohort both acutely when recovery is anticipated, and as a bridge to transplant. Early referral to tertiary heart failure and MCS teams is key to facilitate timely institution of MCS and preserve end-organ function. MCS in children with CHD presents unique challenges due to patient size and complex intra- and extra-cardiac anatomy. Evaluations for MCS should take into account patient size, anatomy, end-organ function, and psychosocial supports. The form of MCS utilized is dependent on clinical urgency, patient size, and anatomy. We describe the evolving landscape of MCS in pediatric patients with CHD.

## 1. Introduction

Pediatric heart failure continues to account for a significant burden of care in children, necessitating prolonged inpatient stays, often in intensive care, and carries an overall in-hospital mortality rate of 6–7% [1,2]. Moreover, congenital heart disease (CHD) accounts for more than half of heart failure admissions to intensive care units (ICUs) in the modern era, with a survival to discharge that is even lower, at 75–81% [3,4,5,6]. With improved early survival outcomes after surgical interventions for CHD, the population of children and young adults living with repaired CHD, often with residual lesions, continues to increase. Despite increasing investment in medical therapies for adults with heart failure related to acquired heart disease, there remains a paucity of medical therapies for heart failure in children, and, in particular, for children with CHD. As such, many progress to end-stage heart failure and require mechanical circulatory support (MCS) to restore cardiac output, provide symptomatic relief, and preserve end-organ function. Mechanical circulatory support includes extracorporeal membranous oxygenation (ECMO) and ventricular assist devices (VADs). MCS can be used as bridge to decision, bridge to transplant, or bridge to recovery. In some cases, VADs are also utilized as chronic or “destination” therapy. Structural heart disease, especially single-ventricle physiology, presents additional technical challenges for the implantation of VADs and in ensuring balanced systemic and pulmonary blood flow. We describe the indications and outcomes of MCS in patients with CHD and delineate the associated challenges in management.

## 2. Indication and Evaluation for Mechanical Circulatory Support

The over-arching indication for MCS in any patient is failure of medical therapy for heart failure in patients with anatomy and physiology amenable to MCS. As such, early recognition of heart failure symptoms in patients with CHD is paramount, as proactive VAD implantation before severe end-organ damage or critical cardiogenic shock improves outcomes [7]. General pediatricians and primary care providers are often the first provider to encounter children with CHD who are developing heart failure and might benefit from VAD therapy and should maintain a high index of suspicion for deteriorating cardiac function. Referral to specialized heart failure and MCS teams should be considered when a young child exhibits persistent feeding difficulties, failure to thrive, respiratory distress, fluid retention, or declining urine output. Similarly in older children, exercise intolerance, evident as fatigue, shortness of breath, or the inability to participate in age-appropriate activities, should prompt referral.

The decision to proceed with MCS should consider patient size, cardiac situs and anatomy, hemodynamic stability, hematologic abnormalities, and extra-cardiac comorbidities that may impact operative risk and capacity for rehabilitation [8]. Whenever possible, semi-elective MCS should be considered in patients with feed intolerance, respiratory impairment requiring mechanical ventilation, or worsening end-organ function (hepatic or renal impairment) or in inotrope-dependent patients unable to complete activities of daily living. For the most part, pediatric patients supported by VADs spend weeks to months in hospital, and as such, a comprehensive psychosocial evaluation should occur prior to implantation, with specific attention to potential barriers to adherence and family support needs [8]. Appropriate timing to institute MCS remains a challenge. It is important to note that improved outcomes are observed in those in whom MCS is instituted prior to the development of significant end organ dysfunction, as evidenced by ventilator dependence or renal impairment. As such, early referral of children with CHD with ventricular dysfunction or heart failure to specialized centres with dedicated heart failure and MCS teams is paramount to achieving optimal outcomes.

## 3. Device Selection

**Figure 1:**
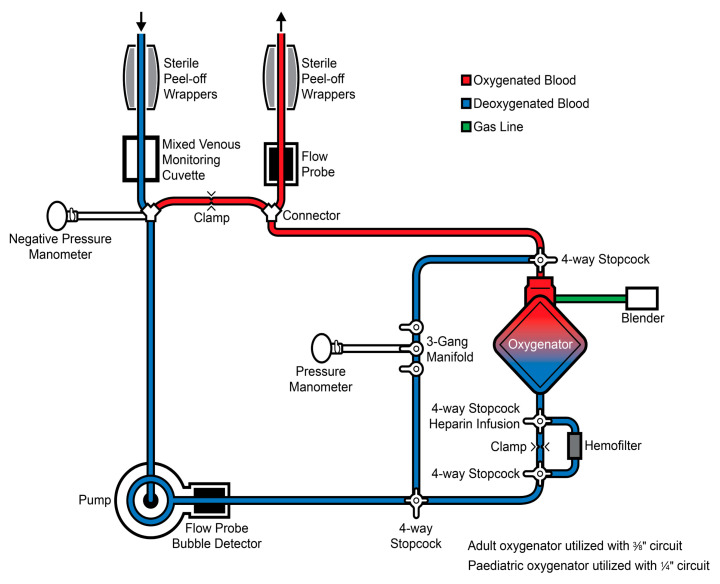
Extracorporeal membranous oxygenation circuit. Image courtesy of the SickKids ECLS Programme.

**Figure 2:**
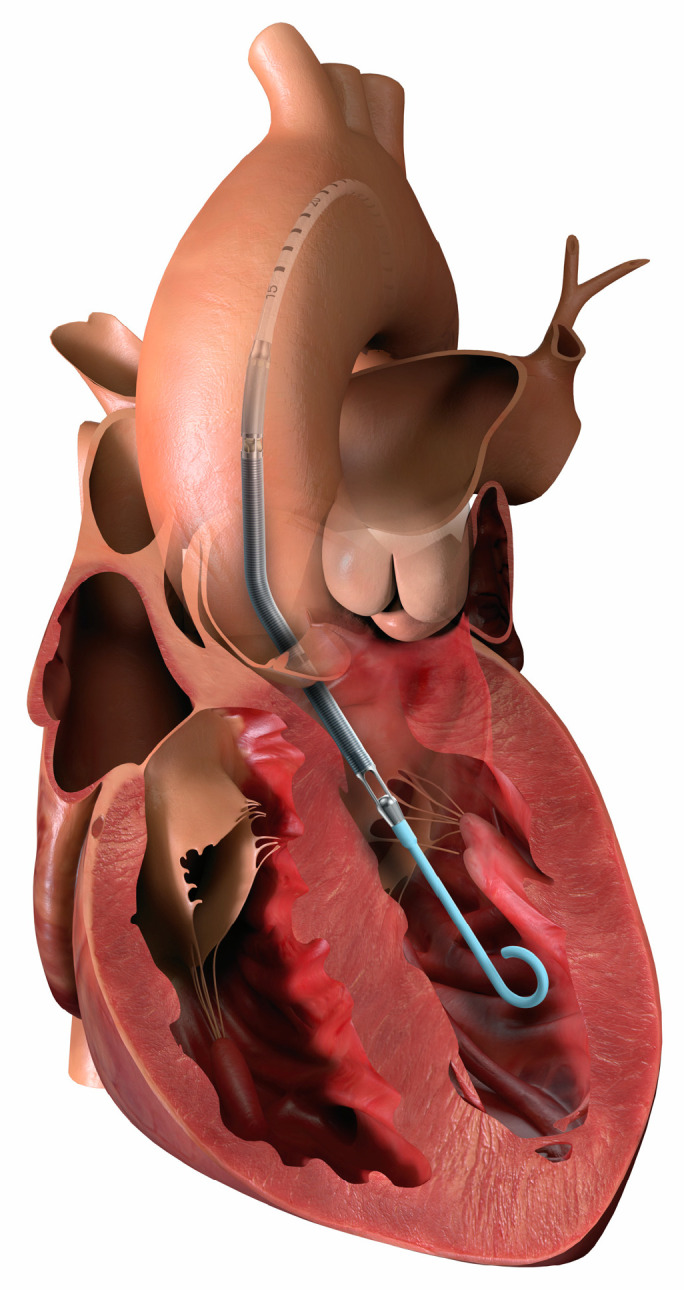
Impella 2.5 device appropriately positioned in the left ventricle across the aortic valve. (Approved by Abiomed Europe GmbH, Aachen, Germany).

Device selection for MCS is contingent on patient size, expected duration of support, hemodynamic stability, device availability, and centre experience. Generally, MCS can be divided into short-term/temporary and durable support strategies. Short-term MCS incorporates ECMO (Figure 1) and Impella (Abiomed, Danvers, MA, USA), a percutaneous microaxial pump (Figure 2). Less commonly used in children or pediatric centres is intra-aortic balloon pumps.

### 3.1. Short-Term Mechanicl Circulatory Support

As distinct from durable VADs, ECMO and Impella should be considered when urgent or emergent support is required, or when clinical improvement over days is anticipated. In these scenarios, ECMO is the most commonly utilized form of support in CHD as it involves cannulation of a main artery and vein and thus can be instituted rapidly in the ICU, and is feasible in most patients irrespective of intra-cardiac anatomy. Central ECMO involves direct cannulation of the right atrium and aorta and is commonly used in cases of failure to wean from cardiopulmonary bypass (CPB) after congenital heart surgery. In peripheral ECMO, neck or groin vessels are used. Peripheral ECMO is more often used in acute presentations and cardiogenic shock, and may need a left heart decompression strategy. It is important to note that as opposed to VADs or Impella, which exclusively provides cardiac output, ECMO circuits also incorporate an oxygenator providing total cardio-pulmonary support. However, the presence of an oxygenator and circuit design significantly limits mobility and increases risk of complications. In most instances, ECMO serves as a bridge to recovery. Where improvement in ventricular function does not occur, patients can be bridged from ECMO to a VAD. While ECMO can be used as a bridge to transplant, recent data have demonstrated markedly higher waitlist and early post-transplant mortality in patients bridged with ECMO and who transitioned from ECMO to a VAD than in those bridged with a VAD alone [8]. Specific indications for ECMO in CHD include intra-operative failure to wean from cardio-pulmonary bypass, pre-operative stabilization, and eCPR. However, recent data demonstrate that patients with CHD listed for transplant are significantly more likely to be supported by ECMO than a VAD while listed, particularly those <10 kg and with single ventricle physiology [8,9]. This likely reflects the additional technical challenges and lack of suitable devices for VAD implantation in small patients with complex anatomy.

Percutaneous microaxial pumps, like the Impella^®^, can be used to provide partial MCS when there is still some native myocardial contribution to cardiac output. There are increasing reports of its use in children and in those with CHD [10]. The major advantage of using a percutaneously deployed pump is that it will obviate the need for a median sternotomy or other invasive cannulation, can be rapidly placed in a cardiac catheterization lab, and can also be used in conjunction with ECMO for left heart decompression (“ecpella”). There is also the option to use it for biventricular short-term MCS (“bipella”).

### 3.2. Durable Ventrivular Assist Devices

Durable VADs can be divided into intracorporeal and paracorporeal devices, and pulsatile and continuous flow devices. The stability and durability of VADs allows for increased mobility and facilitates rehabilitation and engagement with activities of daily living. In addition to the considerations listed above, pre-implant planning should be given to the type of support that is needed. Durable VADs can support the left or systemic ventricle alone (LVAD or SVAD), or if RV support is also needed in a biventricular configuration (BiVAD). The pre-implant workup should include an assessment of RV function and the need for RVAD alongside LVAD. This may involve clinical parameters such as the need for renal replacement therapy, CVP, the etiology of right heart failure, and systolic dysfunction as measured by echocardiography or invasive hemodynamic monitoring [8]. Recent Pedimacs data have demonstrated that as compared with patients without CHD, those with CHD were more likely to be younger at the time of VAD implant (9.8 vs. 5.7, *p* < 0.0001) and more likely to receive a paracorporeal continuous flow or pulsatile device than an implantable continuous flow device [11]. This likely reflects the earlier onset of heart failure in patients with CHD.

#### 3.2.1. Continuous Flow Ventricular Assist Device

**Figure 3:**
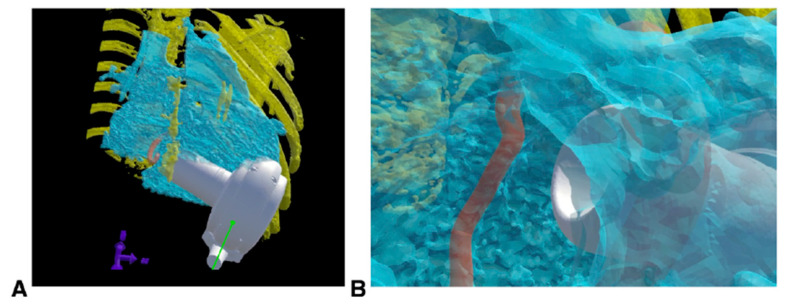
Computed tomography 3D reconstruction of the HeartMate 3™ to assess for device fit within the chest cavity. Image courtesy of Davies et al. 2020, JTCVS Techniques [13]. (**A**) Placement of the ventricular assist device (white) within the reconstructed heart (blue) and ribcage (yellow). (**B**) View of the tricuspid valve annulus (red) and ventricular assist device (white).

The HeartMate 3™ (Abbott, Chicago, IL, USA) intracorporeal continuous flow centrifugal pump is the most commonly used LVAD in adolescents and young adults. For this device, the motor sits inside the thoracic cavity and is connected to an external controller and battery pack via a driveline, which is tunnelled through the skin. Ensuring adequate chest size to fit the motor is essential prior to implantation. Novel techniques using CT imaging with 3D reconstruction have facilitated more accurate assessments of chest size in smaller patients, with the smallest reported patient implant now 19 kg, BSA 0.78 m^2^ (Figure 3). Intracorporeal continuous flow devices have been associated with a lower risk profile and are often manageable by patients and families at home, facilitating greater autonomy and rehabilitation potential while awaiting transplant or recovery. The HeartMate 3™ has been successfully used to support patients with congenital heart disease to transplantation including those with Fontan circulation, with reassuring outcomes [12].

#### 3.2.2. Pulsatile Ventricular Assist Devices

**Figure 4:**
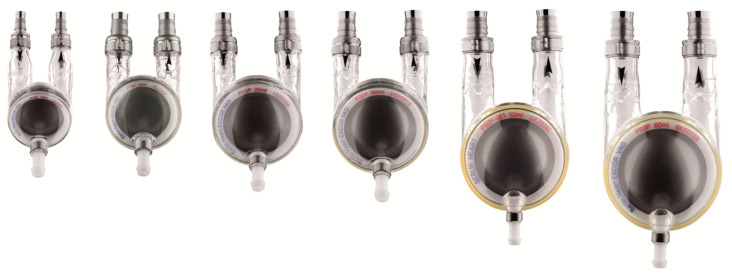
Berlin Heart EXCOR pump sizes; from left to right: 10, 15, 25, 30, 50, and 60 cc pumps. (Courtesy of Berlin Heart GmbH, Berlin, Germany).

While device options remain limited in smaller children, the Berlin Heat EXCOR^®^, a paracorporeal pneumatically driven pulsatile device with tunneled cannulas, continues to be the mainstay of VAD therapy in smaller children, especially those under 20 kg. The devices come with a variety of cannula and pump sizes ranging from 10 cc to 60 cc and has been used in children from their neonatal periods onwards (Figure 4). To date, patients on Berlin EXCOR^®^ have been managed in hospital until transplant or device explant; however, the active driving unit, currently under study, allows for longer battery life, and thus may facilitate greater rehabilitation potential. Some of the inherent benefits of using paracorporeal devices as durable VADs is that they allow for more flexibility in children with CHD and for those with single-ventricle physiology. However, infants and patients under 10 kg remain a high-risk group, with survival to transplant as low as 30% in those with congenital heart disease. This is likely contributed to by technical challenges of VAD implantation in small infants, limited device options, and clinical status at time of VAD implant [8,9]. Thought not available in North America as of yet, Berlin Heart has recently introduced a Fontan cannula to help with the creation of a subpulmonary pump in those with Fontan circulation failure. Others have also created subpulmonary durable VAD strategies, though the experiences are limited to case series [14,15]. Furthermore, Phillip and others reported on their centres’ experiences using paracorporeal pulsatile pumps in infants with hypoplastic left heart syndrome and pulmonary atresia with intact ventricular septum, with an overall survival to discharge of 60% [16,17,18].

#### 3.2.3. Total Artificial Heart

**Figure 5:**
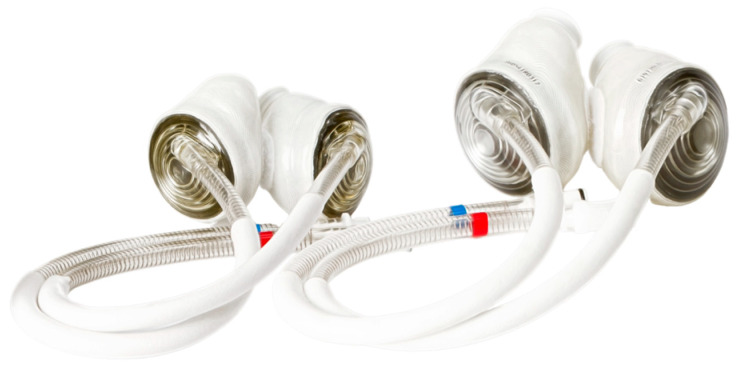
SynCardia™ total artificial heart 70 and 50 cc pumps. Image courtesy of syncardia.com.

**Figure 6:**
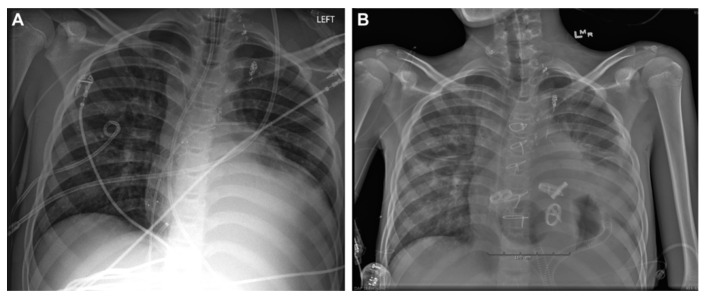
Patient with Fontan circulation implanted with SynCardia™. Image courtesy of Rossano et al. ATS 2014. (**A**) The preoperative and (**B**) postoperative chest x-rays. Note the four artificial valves.

The advent of the SynCardia™ 50 cc total artificial heart (TAH) has provided an additional mechanism for support in patients with complex congenital heart disease with anatomy or physiology not amenable to traditional VAD support (Figure 5 and Figure 6). Implantation of the SynCardia™ involves resection of the ventricular mass and replacement with a biventricular pump and thus is a particularly attractive option in smaller patients in whom anatomy presents technical challenges for systemic VAD implantation. Recent publications have demonstrated increasing VAD use in patients with CHD, in particular smaller and female patients, with a positive outcome achieved in 56%, comparable to those without CHD [18].

## 4. Outcomes and Special Considerations

When compared with those without CHD, patients with CHD supported with VADs have higher mortality rates (36.4% vs. 12.1%, *p* < 0.0001) and lower transplant rates on devices (29.1% vs. 59.9%, *p* < 0.0001) [11]. Patients with CHD also have a greater risk of early respiratory failure on VAD support. However, no significant difference in other major adverse events has been observed, including bleeding, thromboembolism, infection, and neurological dysfunction [11]. For those who survive to transplant on VADs, their post-transplant survival is not different to that of patients without CHD or VAD support. In a recent study, the factors shown to increase mortality risk in patients with CHD undergoing transplant were severe renal impairment, ventilator support, and elevated bilirubin, but VAD support alone did not significantly impact post-transplant survival [19]. Furthermore, VAD support prior to transplant has been associated with a reduction in modifiable risk factors such as end-organ dysfunction with associated improved survival [20].

### 4.1. Biventricular Failure

Despite the high incidence of concomitant RV failure in children with LV dysfunction and increased risk in those with history of CHD affecting the right heart such as Tetralogy of Fallot, the use of biventricular assist devices is relatively low (15%) [21]. In most cases, RV function can be supported on a VAD using a combination of inotropic support; temporary pacing; and pulmonary vasodilator therapies, including inhaled nitric oxide and sildenafil. While several clinical parameters and hemodynamic and imaging measures have been identified to accurately predict RVAD need in adults, evidence in pediatrics is inconclusive. However, RV dysfunction on a VAD is not uncommon and persists in up to 42% [22]. For patients who are deemed to require biventricular support, the most common form of support utilized is biventricular Berlin Hearts. In cases where improvement in RV function is anticipated, temporary RVADs using an extracorporeal continuous flow device may be used.

### 4.2. Systemic RV

Of those with biventricular congenital heart disease, patients with a systemic right ventricle represent a unique cohort. This group largely comprise patients with congenitally corrected transposition of the great arteries (ccTGA) who are at high risk for heart failure due to progressive dilation and dysfunction of the sub-aortic right ventricle [23]. The implantation of VAD in the sub-aortic RV has specific associated challenges, namely the shape of the RV and coarse trabeculations increase the risk of iatrogenic ventricular septal defect during ventricular cannulation. Apical displacement of the tricuspid valve, which is common in ccTGA, may also create difficulties in cannula positioning. Computed tomography with 3D reconstructions can be helpful to aid surgical planning in these scenarios. In some cases, atrio-ventricular (AV) valve repair or replacement may be indicated. Despite the nuances, VAD implantation with a successful bridge to transplant has been described in small pediatric case series [24].

### 4.3. Single-Ventricle Patients

Among CHD patients, survival outcomes are not significantly worse for those with single-ventricle heart disease than biventricular disease. Those with Fontan circulation supported with a VAD have improved survival outcomes on devices than those after Norwood or Glenn palliation (*p* = 0.003) [11]. Patients with Glenn physiology present additional challenges for establishing MCS due to dual systemic venous return and propensity to develop veno-venous collateral with resultant hypoxemia. Some centres advocate for ‘Mechanically assisted Fontan’ with transition to Fontan circulation at the time of VAD insertion due to improved outcomes on a VAD; however, further data are needed to understand if this strategy obviates the increased risk of Stage 2 VAD [14]. Patients undergoing VAD implant prior to the Glenn procedure remain the highest-risk cohort, with mortality rates on a device of 37–46% [18,25]. Up to 80% experience adverse events on a device, the most common of which being major infection.

## 5. Management on Mechanical Circulatory Support

Managing a child on VAD support necessitates a comprehensive, multidisciplinary approach encompassing cardiologists, surgeons, nurses, social workers, and other specialized professionals. Anticoagulation management is a cornerstone of VAD care, requiring a delicate balance between preventing device thrombosis and minimizing bleeding risk. This challenge is amplified in children due to their developmental hemostasis. In most centres, children on pulsatile devices are maintained on Bivalirudin, a direct thrombin inhibitor (DTI) and single or dual antiplatelet therapy. Patients on intracorporeal continuous flow devices are usually initiated on infusion of heparin or DTI and changed to warfarin or oral DTI.

Stringent infection control protocols are essential, as infections, especially driveline infections, are a frequent complication and may lead to systemic complications impacting transplant outcomes. Nutritional support is also of utmost importance, as many children need supplemental enteral or parenteral nutrition to optimize growth and development and enhance post-transplant outcomes. Furthermore, recognizing the significant impact of VADs on the child and family’s quality of life underscores the importance of robust psychosocial support.

## 6. Discussion and Future Prospects

The increased use of MCS in CHD has offered a unique opportunity for supporting patients to transplant and allows for nutrition and rehabilitation such that transplant candidacy and post-transplant outcomes can be improved. While VADs offer substantial improvements in survival and quality of life, they demand specialized expertise and careful patient selection due to the inherent complexity of heart failure in CHD, and thus, early recognition of at-risk patients is essential. Long-term VAD support, while often successful, carries inherent risks. Complications such as bleeding, infection, stroke, and device malfunction can occur, mandating vigilant monitoring, proactive management, and continuous communication with patients and families.

The increasing population of children and young adults with CHD and heart failure has driven innovation in MCS. Adult data have demonstrated survival outcomes on durable VADs such as the HeartMate 3 device that parallel transplant outcomes and, as such, are being utilized as destination therapy in some cases. As the field of MCS evolves and outcomes improve, it is possible that durable VADs such as the HeartMate3 device will be similarly used in pediatric populations, particularly in those with complex congenital heart disease and prior surgeries in whom the presence of HLA antibodies often necessitates more aggressive immunosuppression and carries increased risks.
